# Nutritional and antioxidant dispositions of sorghum/millet-based beverages indigenous to Nigeria

**DOI:** 10.1002/fsn3.140

**Published:** 2014-07-11

**Authors:** Taofeek O Ajiboye, Ganiyat A Iliasu, Abdulwasiu O Adeleye, Folakemi A Abdussalam, Shakirat A Akinpelu, Simiat M Ogunbode, Simiat O Jimoh, Oyelola B Oloyede

**Affiliations:** 1 Antioxidants, Free Radicals, Functional Foods and Toxicology Research Laboratory, Department of Biological Sciences, Al-Hikmah UniversityIlorin, Nigeria; 2 Antioxidants, Free Radicals, and Toxicology Research Laboratory, Biochemistry and Nutrition Unit, Department of Chemical Sciences, Fountain UniversityOsogbo, Nigeria; 3 Nutritional Biochemistry Research Laboratory, Biochemistry and Nutrition Unit, Department of Chemical Sciences, Fountain UniversityOsogbo, Nigeria; 4 Industrial Microbiology Research Laboratory, Department of Biological Sciences, Fountain UniversityOsogbo, Nigeria; 5 Nutritional Biochemistry Research Laboratory, Department of Biochemistry, University of IlorinIlorin, Nigeria

**Keywords:** Lipid peroxidation, *Obiolor*, *Pito*, protein carbonyl, reactive oxygen species

## Abstract

Sorghum/millet-based beverages, *Obiolor* and *Pito*, were evaluated for their nutritional and antioxidant dispositions. Analyzed *Obiolor* and *Pito* contained 96% and 97% moisture; 7.8% and 3.7% crude protein; 8.9% and 5.6% available carbohydrate; 0.39% and 0.31% crude fat; 0.3% and 0.2% crude fiber; 2.4% and 1.5% ash; and 459.3 and 164 kJ/g energy value, respectively. *Obiolor* and *Pito* (1.0 mL) scavenged 2,2-diphenyl-1-picrylhydrazyl by 87% and 81%; superoxide ion by 65% and 59%; hydrogen peroxide by 79% and 76%; and hydroxyl radical by 82% and 85%, respectively. The beverages significantly reduced ferric ion. Aflatoxin B_1_-mediated increase in lipid peroxidation products (conjugated dienes, lipid hydroperoxides, and malondialdehydes) and protein carbonyl in the microsomes were significantly (*P* < 0.05) reduced by the beverages. The data obtained from this study show that the sorghum-based beverages (*Obiolor* and *Pito*) can serve as functional foods, as evident from their antioxidant capabilities in addition to their gross energy content.

## Introduction

Consumer's interests in the health-enhancing roles of specific foods or physiologically active food components (functional foods) have gained rapid increase (Hasler 1998). The physiologically active components of functional foods, which include polyphenols, micronutrients, and macronutrients, proffer physiological benefit beyond basic nutritional requirements (Pang et al. 2012). Functional foods play an important role in the prevention of diseases of metabolic imbalances such as obesity, type 2 diabetes, hypertension, food allergies and intolerances, gastrointestinal and inflammatory disorders as well as cancer. Cereals, vegetables, fruits, pulses, and other plant foods are major sources of antioxidants (e.g., isothiocyanates, phytic acids, flavonoids, phenolics, sterols). Thus, any sorghum/millet-based food products could possess functional food capability. In Nigeria and some other African countries, sorghum/millet is an important component of staple foods.

The nutritional composition of *Sorghum bicolor* grain includes energy (193 cal), moisture content (52%), protein (7.1 g), fat (0.6 g), carbohydrates (39.8 g), fiber (0.9 g), calcium (10 mg), iron (3.5 mg), and niacin (1.7 mg). Phytochemical constituents include phenolics, polyflavonols, thiols, anthocyanins, tannins, 3-deoxyanthocyanidin, flavone, and flavanone (Dykes et al. 2009). Ajiboye et al. (2013a,b) recently reported that *S. bicolor* grains extract protected *N*-nitrosodiethylamine-induced oxidative stress in rat microsomes in vitro and in vivo. Millets are the most widely grown cereals and used in preparation of various kinds of beverages (e.g., koko sour water, *Obiolor*, kunun-zaki) and porridges (koko and kirario). Millet has nutritive value, which resemble that of sorghum. However, pearl millet has slightly lower starch contents, higher protein and lipid content than sorghum and most other common cereals. This makes the energy yield of millet higher than that of sorghum. Millet contains lysine and sulfur containing amino acids, threonine and tryptophan (Matz 1991), and therefore millet has a better amino acid balance than sorghum. Indigenous sorghum-based beverages widely consumed in Nigeria are *Obiolor* and *Pito*.

*Pito* is a light brown alcoholic beverage made from *S. bicolor* grains with sweet–sour taste (Ekundayo 1969). It is widely consumed in Ghana and Nigeria. Traditionally, *Pito* is considered a nutritious source of instant energy for work by many Nigerian tribes. It is also offered to ancestors by pouring it on the ground, a ritual practiced by Nigerians (Tamang and Samuel 2010). It contains lactic acid, sugars, and amino acids and has an alcohol content of 3% (Ekundayo 1969). Organisms responsible for souring include *Geotrichum candidum and Lactobacillus* sp. (Odunfa and Oyewole 1998), while *Candida* sp. are responsible for the alcoholic fermentation. *Pito* is an excellent source of calories and also contributes valuable protein to consumers (Ekundayo 1969).

*Obiolor* is a nonalcoholic beverage produced from fermented sorghum and millet malts in Nigeria. *Obiolor* is consumed daily by the Igala tribe in Nigeria and highly associated with good health. It is a thin gruel with sweet taste. The sweet taste is attributed to sorghum and millet malt (Achi 1990). The microorganisms involved in the fermentation of *Obiolor* are *Lactobacillus plantarum* and *Lactococcus lactis*, which are capable of producing organic acids contributing to the acidity, taste, and aroma of the end product. Presence of *Bacillus* species is also encountered during the fermentation of *Obiolor* (Achi 1990).

Despite the arrays of information available on the nutritional and antioxidant constituents of sorghum and millet, none exist for the sorghum/millet-based beverages (*Obiolor* and *Pito*). This study, thus investigates the nutritional and antioxidant dispositions of *Obiolor* and *Pito*.

## Materials and Methods

### Plant materials

Red variety of *S. bicolor* and millet were obtained from Igbona market, Osogbo, Nigeria. The identification of the sorghum and millet were done at Forestry Research Institute of Nigeria, Ibadan, Nigeria.

### Chemicals and assay kits

Folin–Ciocalteu's reagent, 2,2-diphenyl-1-picrylhydrazyl (DPPH), hydrogen peroxide (H_2_O_2_), iron (II) tetraoxosulfate (IV) (FeSO_4_), 5,5-dimethyl-1-pyrroline-*N*-oxide (DMPO), nitroblue tetrazolium (NBT), phenazine methosulfate (PMS), epicatechin, nicotinamide adenine dinucleotide (NADH), diphenylamine, guanidine hydrochloride, and salicylic acid were procured from Research Organics, Cleveland, OH. All other reagents used were supplied by Sigma-Aldrich Inc., St. Louis, MO.

### Preparation of *Obiolor*

*Obiolor* was prepared in the laboratory using the procedure described by Achi (1990). Briefly, sorghum and millet grains were steeped in water overnight, after which the grains were wrapped in fresh banana leaves and allowed to germinate for 3 days. The germinated grains (80% sorghum + 20% millet) were wet milled and prepared into slurry. The slurry was mixed with boiled water (ratio 1:4 v/v), cooled, filtered, and the residue was discarded, while the filtrate was concentrated by boiling for 30 min with continuous stirring. The resulting gruel was cooled rapidly and allowed to spontaneously ferment for 24 h at ambient temperature and kept frozen.

### Preparation of *Pito*

*Pito* was brewed in the laboratory using the procedure described by Ekundayo (1969). Briefly, sorghum grains were soaked in water for 2 days, followed by malting, and allowing to sit for 5 days in baskets lined with moistened banana leaves. The malted grains were ground, mixed with water, and boiled. The resulting mash was allowed to cool and later filtered through a fine mesh basket. The filtrate obtained was allowed to stand overnight and boiled to a concentrate. A starter from the previous brew was added to the cooled concentrate, which was again allowed to ferment overnight.

### Proximate composition

#### Moisture contents

Moisture was determined using the AACC Method 44–15A (American Association of Cereal Chemists [AACC] 1999). Crude protein was determined using the thermal combustion (Dumas) method with the Leco FP–528 Protein/Nitrogen Analyzer (Leco Corporation, St. Joseph, MI). The nitrogen content was converted to percentage protein by using a protein conversion factor of 6.25. Crude fat was determined by extraction of 3 g of the sample with 40 mL petroleum ether (boiling point 40–60°C) for 4 h according to AACC method 30–25 (1983) using a Soxhlet test apparatus. The AACC method 08–01 (1999) was used to determine the ash content. The total carbohydrate was calculated by difference.

### Gross energy

Gross energy (kJ/g) was estimated by multiplying the percentages of protein, lipid, and carbohydrate by the factors 17, 37, and 16, respectively (FAO/WHO/UNU 1981).

### Quantitative phenolics and flavonoids analysis

#### Total phenolics

The concentrations of phenolic compounds in *Obiolor* and *Pito* were determined using the method described by Spanos and Wrolstad (1991). Briefly, 2.5 mL of 10% Folin–Ciocalteu reagent and 2 mL of Na_2_CO_3_ (2% w/v) were added to 0.5 mL *Obiolor* and *Pito*. The resulting mixtures were incubated at 45°C with constant shaking for 15 min. The absorbance of the samples was read at 765 nm. This was done in triplicate. The total phenolic content in the beverages was expressed as mg of epicatechin (0–0.5 mg/mL) dissolved in distilled water.

#### Total flavonoids

The concentration of total flavonoids in the beverages (*Obiolor* and *Pito*) was determined using the procedure described by Zhishen et al. (1999). Briefly, *Obiolor* and *Pito* (1 mL each) was mixed with 3 mL of methanol, 0.2 mL of 10% aluminum chloride, 0.2 mL of 1 mol/L potassium acetate, and 5.6 mL of distilled water. The mixture was allowed to stand at room temperature for 30 min. The absorbance of the reaction mixture was read at 420 nm. The concentration of flavonoids in mg/mL was obtained from the calibration curve of epicatechin solution (0–0.8 mg/mL) in distilled water.

### Free radical and reactive oxygen species scavenging assays

#### DPPH radical scavenging assay

The antioxidant activities of *Obiolor* and *Pito* were determined by measuring the capacity of bleaching a purple-colored ethanol solution of DPPH, as described by Turkoglu et al. (2007), with a slight modification. Briefly, 2 mL of various concentrations (20–100%) of the samples in ethanol were added to 2 mL of 0.2 mmol/L sample of DPPH in ethanol. After 30-min incubation period at room temperature, the absorbance was read against blank at 517 nm. Inhibition rate (%I) on the DPPH radical was calculated using the expression:


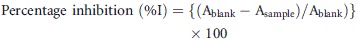


### Superoxide anion radical (O_2_^•−^) scavenging assay

The scavenging effects of *Obiolor* and *Pito* on superoxide anion radical were examined by the spectrophotometric measurement of the product formed on reduction in NBT (Yen and Chen 1995). Briefly, superoxide anion was generated in a nonenzymic system. The reaction mixture contained 1 mL of the beverages (20–100% v/v) in distilled water, 1 mL of 60 *μ*mol/L of PMS in phosphate buffer (0.1 mol/L, pH 7.4), 1 mL of 468 *μ*mol/L of NADH in phosphate buffer, and 1 mL of 150 *μ*mol/L of NBT in phosphate buffer and was incubated at ambient temperature for 5 min, and the color was read at 560 nm against blank samples.

### Hydrogen peroxide scavenging assay

Hydrogen peroxide (H_2_O_2_) scavenging activities of *Obiolor* and *Pito* were determined as described by Ruch et al. (1989). Briefly, 3.4 mL of varying concentrations (20–100% v/v) of *Obiolor* and *Pito* were mixed with 0.6 mL H_2_O_2_ (40 mmol/L). The absorbance was read at 230 nm after 10 min of incubation at room temperature. The percentage H_2_O_2_ scavenging activities of *Obiolor* and *Pito* were calculated using the following expression:


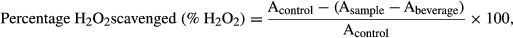


where A_control_ is the absorbance of the mixture without beverage, A_sample_ is the absorbance of the mixture with the beverage, and A_beverage_ is the absorbance of the beverage alone.

### Hydroxyl radical (OH^•−^) scavenging assay

OH^•−^ scavenging activities of *Obiolor* and *Pito* were measured as described by Smirnoff and Cumbes (1989) with slight modifications. Briefly, 2 mL of *Obiolor* and *Pito* at concentrations ranging from 20% to 100%, 0.6 mL of 8 mmol/L ferrous sulfate, 0.5 mL of 20 mmol/L hydrogen peroxide, and 2 mL of 3 mmol/L salicylic acid were mixed and incubated at 37°C for 30 min. Then, 0.9 mL of distilled water was added to each vial. The final solution was centrifuged at 4472 *g* for 10 min. After centrifugation, the absorbance was measured at 510 nm. The percentage OH^•−^ scavenging activities of *Obiolor* and *Pito* were calculated using the following expression:


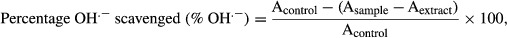


where A_control_ is the absorbance of the mixture without beverage, A_sample_ is the absorbance of the mixture with the beverage, and A_beverage_ is the absorbance of the beverage alone.

### Reducing power

The reducing power of *Obiolor* and *Pito* was evaluated by adopting the method of Oyaizu (1986). Varying concentrations of the beverages (20–100%) were suspended in 1 mL of distilled water and mixed with 2.5 *μ*L of 0.2 mol/L phosphate buffer (pH 6.6) and 2.5 mL of 1% potassium ferricyanide [K_3_Fe(CN)_6_]. The mixture was incubated at 50°C for 20 min, after which 2.5 *μ*L of trichloroacetic acid (TCA) was added to the mixture. Following centrifugation at 402 *g* for 10 min, 2.5 *μ*L of the supernatant was mixed with an equal amount of distilled water and 0.5 mL of 0.1% FeCl_3_. The absorbance of the resulting solution was measured at 700 nm.

### Inhibition of reactive oxygen species generation, lipid peroxidation, and protein oxidation

#### Preparation of hepatic microsomes

Liver was homogenized in sucrose–Tris buffer (0.25 mol/L sucrose, 10 mmol/L Tris–HCl, pH 7.4) as described by Ajiboye et al. (2014). Liver microsomes were isolated by the calcium chloride precipitation method described by Kondraganti et al. (2005). The protein concentration of the microsomal fraction was determined according to Gornall et al. (1949) and the microsomes were finally stored at −80°C.

### Induction of microsomal lipid peroxidation and protein oxidation

Oxidation of microsomal lipids and proteins was done as described by Ajiboye et al. (2013a) with little modifications. The reaction mixture consisted of 1.25 mg microsomal proteins, 0.1 mol/L MgCl_2_, 0.2 mol/L bovine serum albumin (BSA), 2 mmol/L NADPH, and AFB_1_ with or without *Obiolor*, *Pito*, or vitamin C in sucrose–Tris buffer (0.25 mmol/L sucrose, 10 mmol/L Tris-HCl, pH 7.4). The incubation was carried out for 1 h at 37°C with intermittent shaking and the reaction was terminated by chilling the mixture on ice after which the resulting mixtures were assayed for superoxide anion radical, lipid peroxidation products, and protein carbonyl.

### Reactive oxygen species (superoxide anion radical) production

The generation of ROS by cells (respiratory burst) was measured by the formation of colored formazan due to reduction in NBT. Briefly, appropriately diluted microsomes from incubation mixtures (final volume 1 mL) was mixed with 1 mL of 150 *μ*mol/L of NBT in phosphate buffer and was incubated at ambient temperature for 5 min, and the color was read at 560 nm.

### Lipid peroxidation and protein oxidation assay

The levels of lipid peroxidation products (conjugated dienes, lipid hydroperoxides, and malondialdehyde) in the microsomes were determined using the procedures described by Bus et al. (2001). Protein carbonyl, a marker of protein oxidation, was assessed in the microsomes using the procedure described by Levine et al. (1990).

### Statistical analysis

All experimental values were represented as mean ± SD (*n* = 3). Free radical and ROS scavenging activities were expressed in percentage. Analysis of variance (ANOVA) followed by Tukey–Kramer test for differences between means was used to account for any significant differences (*P* < 0.05) between the variables in this study using StatPlus, 2011 (AnalystSoftInc, Alexandria, VA).

## Results and Discussion

Wise use of fruits, medicinal plants, and vegetables requires investigations into the phytochemicals and antioxidants as well as the possible medicinal properties and prospective products, such as nutraceuticals and phytomedicines (Oloyede et al. 2013). This study provides the nutritional and antioxidants disposition of sorghum/millet-based beverages (*Obiolor* and *Pito*).

### Proximate composition

Proximate analysis is an important tool in the evaluation of nutritional status of foods and food products. The proximate and nutritional composition of *Obiolor* and *Pito* are presented in Table 1. The very high level of moisture content of the beverages (Table 1) is expected, as they are liquid-based beverages. Thus, the beverages would be better stored airtight and in refrigerator. Protein is an essential macronutrient for growth and maintenance of body tissues (WHO/FAO Report 1985). The protein content of sorghum grains is within the range of 5.44–12.9% (Salinas et al. 2006). The level of protein in the beverages (Table 1) indicates its potential to complement protein-based meal. The higher protein content of *Obiolor* could be due to the millet component of the beverage, as millet is a better source of protein than sorghum.

**Table 1:** Nutritional composition of sorghum/millet-based beverages.

Nutrients	*Obiolor*	*Pito*
Moisture content (%)	80.21 ± 0.24	88.69 ± 0.48
Crude protein (%)	7.80 ± 0.12	3.70 ± 0.02
Available carbohydrate (%)	8.90 ± 0.05	5.60 ± 0.31
Crude fat (%)	0.39 ± 0.01	0.31 ± 0.01
Crude fiber (%)	0.30 ± 0.01	0.20 ± 0.01
Ash (%)	2.40 ± 0.03	1.50 ± 0.01
Energy value (kJ/g)	459.30 ± 0.42	164.00 ± 3.12

Available carbohydrates are digestible, absorbable, and hence utilizable nutritionally (Oloyede 2005). These include sugars like glucose, fructose (found in fruits, vegetables, honey, and sugarcane), starch (which is the storage form of carbohydrates in plants and contains amylopectin and amylose) as found in cereals, and tubers like yam, cassava, potatoes, etc. (Oloyede 2005). Thus, the appreciable amount of available carbohydrates in the beverages (Table 1) could serve as source of energy derivable as adenosine triphosphate. The low level of unavailable carbohydrates (dietary fibers) is not a surprise, as majority of the fibers (pectin, cellulose, hemicellulose, and lignin) in the sorghum and millet could have been hydrolyzed to simple sugar during fermentation.

Gross energy (kJ/g) establishes the relationship between food composition and available energy (FAO/WHO/UNU 1981). The gross energy (kJ/g) of *Obiolor* is appreciable higher than that of *Pito* (Table 1). This could have resulted from the lower amount in the nutritional composition of *Pito* brought about by longer day of fermentation. Thus, *Obiolor* is a better source of energy.

### Free radical and reactive oxygen species scavenging activities

**Figure 1:**
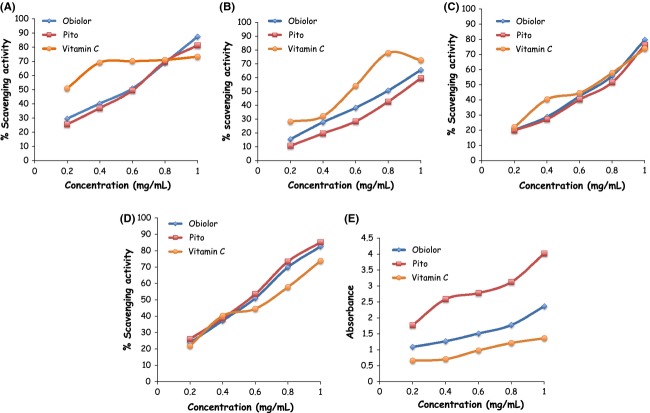
(A) DPPH scavenging; (B) superoxide anion radical scavenging; (C) hydrogen peroxide scavenging; (D) hydroxyl radical scavenging; and (E) reducing activity of the polyphenolic extract of *Obiolor* and *Pito* beverages.

Free radicals are highly reactive substances capable of giving rise to chain reactions, that is, reactions that involve a number of steps, each of which forms a free radical that triggers the next step. Studies have indicated the free radical scavenging properties of sorghum extracts and sorghum-based products in vitro (Awika et al. 2003; Ajiboye et al. 2013a). Thus, the DPPH radical scavenging activity produced by *Obiolor* and *Pito* (Fig. 1A) indicates hydrogen ion donating capability of the beverages. This could be important in the prevention of free radical-induced lipid peroxidation.

ROS (^•^OH, O_2_^•−^, and H_2_O_2_) scavenging activities of *Obiolor* and *Pito* (Fig. 1B–D) show potentials of the beverages in complementing ROS detoxifying enzymes in vivo, if effectively absorbed. It could also help terminate lipid peroxidation that could arise from O_2_^•−^, and the stronger ROS (O^•^,^•^OH and H_2_O_2_) (Yen and Der Duh 1994). Also, the capability of beverages to significantly reduce K_3_Fe(CN)_6_ (Fig. 1E) indicates effectiveness to halt oxidation of cellular macromolecules by oxidizing molecules that could arise from the metabolism of either drugs or toxins. The free radical and ROS scavenging activity recorded in this study could be due to the high level of flavonoids and phenolics present in the beverages (Table 2).

**Table 2:** Total phenols and total flavonoids content of sorghum/millet-based beverages.

	Total phenols	Total flavonoids
*Obiolor*	582 ± 2.50	537 ± 7.81
*Pito*	310 ± 1.06	275 ± 0.70

The results are mean ± SD for three replicates.

ROS production in the AFB_1_-treated microsomes increased significantly (*P* < 0.05) when compared with no treatment (Table 3). *Obiolor* and *Pito* significantly reduced AFB_1_-mediated generation of ROS as does by the reference antioxidants, vitamin C and compared with the control (Table 3).

**Table 3:** Levels of lipid peroxidation products, protein carbonyl, and superoxide anion radical following treatment of sorghum/millet-based beverages to aflatoxin B_1_-treated microsome.

Treatments	Conjugated dienes (nmol mg protein^−1^)	Lipid hydroperoxide (nmol mg protein^−1^)	Malondialdehyde (nmol mg protein^−1^)	Protein carbonyl (nmol mg protein^−1^)	Superoxide anion radical (*μ*mol NBT reduce mg protein^−1^)
Untreated microsomes	51.58 ± 0.86^a^	49.81 ± 0.45^a^	5.69 ± 0.43^a^	4.81 ± 0.11^a^	3.26 ± 0.52^a^
AFB_1_-treated microsomes	118.59 ± 3.51^b^	123.92 ± 0.48^b^	15.70 ± 0.23^b^	8.05 ± 0.18^b^	13.23 ± 0.42^b^
*Obiolor*	51.86 ± 1.26^a^	50.23 ± 0.60^a^	5.62 ± 0.18^a^	4.18 ± 0.03^a^	3.23 ± 0.12^a^
*Pito*	50.98 ± 1.34^a^	47.83 ± 0.15^a^	5.40 ± 0.21^a^	4.61 ± 0.02^a^	3.83 ± 0.30^a^
*Obiolor* + aflatoxin B_1_	59.24 ± 1.06^c^	57.42 ± 0.12^c^	6.49 ± 0.28^c^	4.17 ± 0.03^a^	6.22 ± 0.24^c^
*Pito* + aflatoxin B_1_	55.42 ± 0.61^c^	58.27 ± 0.70^c^	6.22 ± 0.46^c^	4.54 ± 0.05^a^	7.27 ± 0.70^c^
Vitamin C + aflatoxin B_1_	52.58 ± 0.84^a^	59.01 ± 0.26^c^	5.89 ± 0.32^a^	4.76 ± 0.04^a^	5.01 ± 0.12^c^

The results are mean ± SD for three replicates. Values carrying superscripts different for each parameter are significantly different (*P* < 0.05).

### Lipid peroxidation and protein oxidation

Lipid peroxidation, which is constantly taking place in vivo, induces disturbances of membrane organization and functional loss and modification of proteins and DNA bases (Niki 2009). AFB_1_ has been reported to induce lipid peroxidation in numerous in vitro and in vivo methods of carcinogenesis (Shen et al. 1994; Towner et al. 2002; Lee et al. 2005; Theumer et al. 2010; Ravinayagam et al. 2012). Thus, the significant increase in the levels of lipid peroxidation products (conjugated dienes, lipid hydroperoxides, and malondialdehydes) (Table 3) shows indiscriminate oxidative assaults on the cellular lipids. These increases (most especially conjugated dienes) could result to mutation (Das et al. 2010). The capability of the beverages to reverse AFB_1_-mediated increases in conjugated dienes, lipid hydroperoxides, and malondialdehyde may be due to the ROS scavenging activity of the beverages. It may also be due to the capability of polyphenolic components of *S. bicolor* to promote ROS detoxification through the induction of antioxidant enzymes (Ajiboye et al. 2013b), which could cause the peroxidation of polyunsaturated fatty acids of plasma membrane.

Protein carbonyl is an indicator of irreversible damage to cellular proteins and may have lasting detrimental effects on cells and tissues (Dalle-donne et al. 2003). Thus, the significant increase in protein carbonyl in AFB_1_-treated microsomes (Table 3) could have resulted from the oxidation of protein by free radicals and ROS generated during AFB_1_ metabolism. The attenuation of AFB_1_-mediated increase in the level of protein carbonyl by *Obiolor* and *Pito* further shows possible ROS scavenging and capability to halt oxidative onslaught on protein, possibly through ROS detoxification. Similar attenuation of AFB_1_-mediated increase in protein carbonyl level following the administration of Tridham has been reported (Ravinayagam et al. 2012).

## Conclusion

It can be deduced from this study that the sorghum-based beverages (*Obiolor* and *Pito*) can serve as functional foods, as evident from the gross energy contents, antioxidant activities, and capabilities to prevent the AFB_1_-mediated oxidation of lipids and proteins. Thus, the consumption of these beverages is encouraged as functional foods.
